# Detection Rate of Prostate Cancer in Repeat Biopsy after an Initial Negative Magnetic Resonance Imaging/Ultrasound-Guided Biopsy

**DOI:** 10.3390/diagnostics13101761

**Published:** 2023-05-17

**Authors:** Magdalena Görtz, Ann-Kathrin Huber, Tim Linz, Constantin Schwab, Albrecht Stenzinger, Lukas Goertz, David Bonekamp, Heinz-Peter Schlemmer, Markus Hohenfellner

**Affiliations:** 1 Department of Urology, University Hospital Heidelberg, 69120 Heidelberg, Germany; markus.hohenfellner@med.uni-heidelberg.de; 2 Junior Clinical Cooperation Unit ‘Multiparametric Methods for Early Detection of Prostate Cancer’, German Cancer Research Center (DKFZ), 69120 Heidelberg, Germany; 3 Medical Faculty, Ruprecht-Karls University of Heidelberg, 69117 Heidelberg, Germany; ann-kathrin.huber@stud.uni-heidelberg.de (A.-K.H.); timlinz@web.de (T.L.); 4 Institute of Pathology, University of Heidelberg, 69120 Heidelberg, Germany; constantin.schwab@med.uni-heidelberg.de (C.S.); albrecht.stenzinger@med.uni-heidelberg.de (A.S.); 5 Department of Radiology, Medical Faculty and University Hospital, University of Cologne, 50939 Cologne, Germany; lukas.goertz@uk-koeln.de; 6 Divison of Radiology, German Cancer Research Center (DKFZ), 69120 Heidelberg, Germany; d.bonekamp@dkfz-heidelberg.de (D.B.); h.schlemmer@dkfz-heidelberg.de (H.-P.S.)

**Keywords:** MRI/TRUS fusion biopsy, magnetic resonance imaging, PI-RADS, prostate cancer, prostate-specific antigen

## Abstract

A negative multiparametric magnetic resonance imaging (mpMRI)-guided prostate biopsy in patients with suspected prostate cancer (PC) results in clinical uncertainty, as the biopsy can be false negative. The clinical challenge is to determine the optimal follow-up and to select patients who will benefit from repeat biopsy. In this study, we evaluated the rate of significant PC (sPC, Gleason score ≥7) and PC detection in patients who received a follow-up mpMRI/ultrasound-guided biopsy for persistent PC suspicion after a negative mpMRI/ultrasound-guided biopsy. We identified 58 patients at our institution that underwent repeat targeted biopsy in case of PI-RADS lesions and systematic saturation biopsy between 2014 and 2022. At the initial biopsy, the median age was 59 years, and the median prostate specific antigen level was 6.7 ng/mL. Repeat biopsy after a median of 18 months detected sPC in 3/58 (5%) patients and Gleason score 6 PC in 11/58 (19%). Among 19 patients with a downgraded PI-RADS score at the follow-up mpMRI, none had sPC. In conclusion, men with an initial negative mpMRI/ultrasound-guided biopsy had a high likelihood of not harboring sPC at repeat biopsy (95%). Due to the small size of the study, further research is recommended.

## 1. Introduction

Prostate cancer (PC) is the second most frequently diagnosed malignancy in men, representing 27% of all new cancer diagnoses in this group [1]. Early detection of PC through screening has several advantages, including a higher likelihood of cure, the availability of less invasive treatment options, a reduced risk of advanced or metastatic disease, and improved quality of life [2,3,4]. Biomarkers such as prostate-specific antigen (PSA) and a digital rectal examination (DRE) can be used as screening methods to facilitate the early detection of PC. However, PSA testing lacks specificity, as it is elevated not only in PC, but also in prostatic hyperplasia and prostatitis, resulting in unnecessary prostate biopsies [5]. Complications such as bleeding, infection, and urinary retention can arise from biopsies, resulting in increased burden and costs [6,7]. To accurately assess a patient’s risk of tumor progression, the prostate biopsy result is commonly used to stratify patients into significant PC (sPC) and indolent PC, which potentially will not become clinically relevant to the patient [8]. In the case of indolent PC, active surveillance protocols have been included as an acceptable choice and as a management option to delay the side effects of treatment without missing tumor progression to aggressive PC [9]. Different studies use varying definitions of PC that requires definite treatment. One common definition of sPC is the detection of a Gleason score (GS) of ≥3 + 4 in the biopsy cores [10,11].

Multiparametric magnetic resonance imaging (mpMRI) and fusion biopsy have emerged as promising techniques for PC detection, as they enhance the accuracy of PC diagnosis [12,13,14,15,16]. MpMRI helps to reduce the under-detection of aggressive PC and the overdiagnosis of insignificant PC [12]. Pre-biopsy risk assessment with mpMRI followed by mpMRI-targeted fusion biopsy (TB) has been shown to be more effective than the standard transrectal ultrasonography (TRUS)-guided prostate biopsy [13]. In this context, recent level I trials have challenged the standard 12-core TRUS-guided biopsy as the standard for PC detection [12,13,14,15,16]. MpMRI has been recommended for routine use before prostate biopsy in biopsy-naïve patients due to its superior detection rates of sPC [12,13,15,16]. In patients with suspected PC, mpMRI of the prostate is now considered mandatory prior to prostate biopsy [17].

The Prostate Imaging Reporting and Data System (PI-RADS) classification, proposed by the American College of Radiologists and European Society of Urogenital Radiology, has been suggested for standardized MRI interpretation [18,19]. The PI-RADS classification, first published in 2012, has been revised twice [19,20]. This classification system scores mpMRI lesions on a scale of 1 to 5 based on the likelihood of PC, with higher PI-RADS scores indicating a greater probability of PC [21].

When a patient has a PI-RADS 4/5 lesion on initial mpMRI, a negative mpMRI/TRUS-guided biopsy presents a clinical challenge for future patient management [22]. A negative initial biopsy may be due to either a false positive mpMRI result or a false negative biopsy [23]. In a Cochrane systematic review, mpMRI had a high sensitivity of 0.91 for the detection of sPC, but a limited specificity of 0.37 for the diagnosis of sPC [16]. A recent meta-analysis showed that the positive predictive value of an mpMRI lesion classified as PI-RADS ≥ 3 was approximately 40%, reaching a value of 69% in men receiving targeted-fusion biopsy for PI-RADS 5 lesions [24]. Thus, a considerable number of patients with a positive mpMRI do not harbor sPC. While the presence of PI-RADS 4/5 lesions on prostate mpMRI often correlates with PC, there are several benign histopathological findings that mimic suspicious lesions on MRI [22]. Otherwise, a false negative biopsy can be due to targeting error and misregistration, which can result from deformation of the prostate gland or errors in prostate segmentation. In addition, PC detected on repeat biopsy may have been missed on the initial biopsy or may have arisen de novo due to disease progression [25]. To date, there is a lack of evidence and international recommendations to support a specific follow-up strategy in men with a negative mpMRI/TRUS-guided biopsy. In clinical routine, the decision to repeat mpMRI and/or prostate biopsy, or to measure PSA alone, is at the clinician’s discretion [26].

This study evaluates the rate of sPC and PC detection in repeat mpMRI/TRUS-guided biopsy after an initial negative mpMRI/TRUS-guided biopsy. At the same time, we aimed to identify subgroups of patients with a negative initial prostate biopsy that have a negligible risk of harboring sPC and who could therefore potentially avoid a further biopsy. This would provide clinicians with useful information on how to follow-up patients with an initial negative mpMRI/TRUS-guided fusion biopsy without missing men at higher risk of sPC.

## 2. Materials and Methods

### 2.1. Study Population

**Figure 1:**
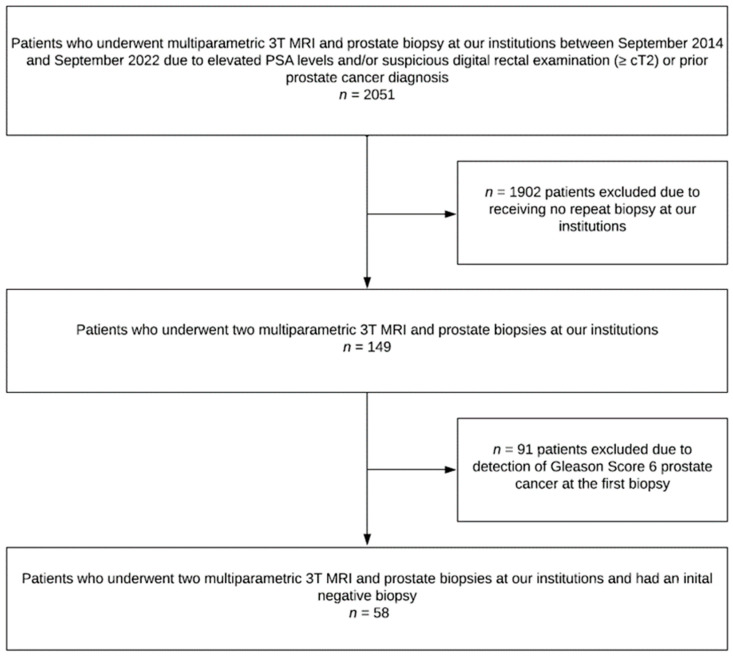
Flow chart for study inclusion. MRI = multiparametric imaging; PSA = prostate-specific antigen.

We conducted a retrospective analysis of 2051 cases in which patients underwent mpMRI and combined targeted (in the case of a suspicious PI-RADS lesion) and systematic prostate biopsy at our institutions between September 2014 and September 2022. Of these cases, 58 men underwent two mpMRI/TRUS-guided biopsies at the University Hospital in Heidelberg, Germany, because of a persistent suspicion of PC after a negative initial biopsy (Figure 1). At the time of the first biopsy, all 58 men had a PSA level of ≥4 ng/mL and/or suspicious DRE and no previous diagnosis of PC. As there are no standardized follow-up protocols for negative initial MRI/TRUS-guided biopsies, the follow-up was completely up to the decision of the referring outpatient urologist and patient. A repeat biopsy was performed if suspicion of PC remained based on individual considerations such as high or rising PSA, as well as persistent or increasing abnormalities on mpMRI. The mpMRIs were performed on a 3 Tesla MRI system (Magnetom Prisma, Siemens Healthcare, Erlangen, Germany) at the German Cancer Research Center. Complete clinical data, including age, PSA, prostate volume, PI-RADS score (≤2 vs. 3 vs. 4 vs. 5), number of biopsy cores, and GS assigned at the initial and repeat biopsy, were available for all patients in the cohort. Data were collected prospectively, and institutional review board approval was obtained (S-164/2019 and S-130/2021). This study includes previously published patient data [27,28,29].

### 2.2. MRI Analysis

Prebiopsy mpMRI without the use of an endorectal coil was performed in all patients. MRI was performed with a 3-T scanner (MAGNETOM Prisma, Siemens Healthineers, Erlangen, Germany). All mpMRI scans followed the PI-RADS recommendations and were acquired with a standard multichannel body coil and an integrated spinal phased array coil, according to European Society of Urogenital Radiology guidelines [19,30]. The imaging protocol included axial, coronal, and sagittal T2-weighted images; echo-planar diffusion-weighted images (DWI) with apparent diffusion coefficient (ADC) maps; and dynamic contrast-enhanced images (Supplementary Material S1). DWI was performed using b-values of 50, 500, 1000 and 1500 s/mm^2^. Dynamic contrast-enhanced MRI was performed with a T1-weighted transverse time-resolved MR sequence (VIBE or VIBE TWIST; fat-saturated) during the bolus injection of a standard weight-adapted dose of 0.1 mmol/kg bodyweight of intravenous Gadobutrol (Gs-BT-DO3A, Bayer Vital, Leverkusen, Germany) at an injection rate of 3 mL/s, followed by a 50 cc sodium chloride flush. MpMRI reporting was accomplished as part of clinical routine by board-certified radiologists (each with more than three years of experience in prostate imaging), according to PI-RADS guidelines. All exams were reviewed in a multidisciplinary conference prior to biopsy for quality assurance, including a review of all lesion targets stated in the clinical reports. The exams in the study cohort period were assessed under the supervision and guidance of the same specialized uro-radiologist with longstanding experience in prostate imaging (DB). In addition, all radiologists retrospectively reviewed the mpMRI reports and biopsy results on a regular basis.

### 2.3. Biopsy

All patients underwent a transperineal prostate biopsy while under general anesthesia. The biopsies were performed using either the MedCom BiopSee platform (MedCom, Darmstadt, Germany) or UroNav (Philips Invivo, Gainsville, FL, USA), which utilize software registration. Prostate biopsy cores were taken by an 18 G × 250 mm biopsy needle with the assistance of a bk medical flex focus 1202 ultrasound device and a 12–4 MHz ultrasound frequency range. The biopsy procedure involved TB in case of suspicious lesions identified on the MRI, followed by a systematic saturation biopsy (SB) in accordance with the Ginsburg protocol, as described in previous literature [10,11,31]. At our institution, SB is performed with an increased number of biopsy cores to ensure maximum diagnostic safety for patients. The combined transperineal SB and TB biopsy approach has been validated and confirmed to be in accordance with radical prostatectomy (RP) samples [31]. Only urologists with significant experience, having performed > 50 mpMRI/TRUS fusion biopsies, conducted the prostate biopsies in our study cohort. Men without a PI-RADS ≥ 3 lesion on mpMRI underwent transperineal SB.

### 2.4. Pathology

The targeted and systematic core samples were securely fixed in paraffin and subjected to separate analysis under the guidance of a specialized uro-pathologist (AS), adhering to the standards set by the International Society of Urological Pathology (ISUP) [32]. The diagnosis of sPC was made based on a GS of 3 + 4 or higher.

### 2.5. Statistical Analysis

Cohort characteristics were summarized using descriptive statistics, including median and interquartile range (IQR) for continuous variables and frequencies for categorical variables. Patient characteristics were compared between those who had PC detected by repeat biopsy and those who did not, using *t*-tests for continuous variables and chi-squared tests/Fisher’s exact tests for categorical variables. All statistical analyses were performed at a 5% significance level.

## 3. Results

### 3.1. Patient Characteristics at Initial Prostate Biopsy

The study cohort consisted of 58 patients who received a subsequent MRI/TRUS-guided prostate biopsy after an initial negative MRI/TRUS-guided biopsy. At the initial biopsy, the median age was 59 (IQR: 54–64) years and the median initial PSA was 6.7 (IQR: 4.8–8.9) ng/mL. The median prostate volume was 53 (IQR: 41–70) ml and the median number of sampled biopsy cores was 31 (IQR: 27–33). Overall, a PI-RADS score of 1–2 was present in 7 patients, PI-RADS 3 in 19 patients, PI-RADS 4 in 26 patients, and PI-RADS 5 in 6 patients. We identified a group of 14 patients (24%) in whom PC was detected on the repeat biopsy, and a group of 44 patients (76%) who showed no malignancy on the repeat biopsy. The two groups did not differ in clinical parameters such as age or PSA at the time of the initial biopsy, as shown in Table 1. The two groups differed in the number of PI-RADS 3 lesions on the initial MRI (*p* = 0.03), but the number of PI-RADS 3–5 lesions on the initial MRI did not differ between the two groups (*p* = 0.11).

### 3.2. Patient Characteristics at Repeat Prostate Biopsy

The median time between the initial and repeat biopsy was 18 (IQR: 10–24) months (Table 2). sPC detection at the repeat biopsy was 5% (3/58). Of the 14/58 patients who had PC detection at the repeat biopsy, seven patients had PC detection in the TB, three patients only had PC detection in the SB, and four patients only had PC detection in the SB due to a negative MRI. In 9/14 patients with PC detection at the repeat biopsy, the lesion on the repeat MRI was seen in the same location as on the initial MRI, in 1/14 patient a new suspicious lesion was seen on repeat MRI, and in 4/14 patients the repeat MRI was without suspicious lesion. At the repeat biopsy, the median age of the entire cohort was 61 (IQR: 56–69) years and the median PSA increased to 8.0 (IQR: 5.5–11.8) ng/mL. The median prostate volume was 60 (IQR: 47–88) ml and the median number of sampled biopsy cores was 31 (IQR: 26–35). Overall, a PI-RADS score of 1–2 was present in 11 patients, PI-RADS 3 in 22 patients, PI-RADS 4 in 19 patients, and PI-RADS 5 in 6 patients. A downgrading of PI-RADS score on the follow-up mpMRI occurred in 33% (19/58) of patients and upgrading in 16% (9/58). The PI-RADS score remained persistent on the repeat mpMRI in 47% (27/58) of patients, while mpMRI remained negative in 5% (3/58) of patients. Four of the patients with PI-RADS score downgrading on the follow-up mpMRI had PC on the repeat biopsy, but none had sPC. The two groups (PC detection vs. no malignancy on repeat biopsy) were similar in terms of clinical parameters at the time of the repeat biopsy, as shown in Table 2. For example, there was no significant difference between PSA level and the risk of PC detection at the repeat biopsy that could be used to decide for or against the procedure. Similarly, there was no association between the presence of a PI-RADS 3–5 lesion or the persistence/upgrading of a PI-RADS lesion on the repeat mpMRI and the detection of PC at the repeat biopsy (Table 2). When comparing PI-RADS 3–5 lesions to PI-RADS 1–2 lesions on the repeat mpMRI for predicting PC detection at the repeat biopsy, the sensitivity was 0.71, specificity 0.16, positive predictive value 0.21, and negative predictive value 0.64. When comparing the downgrading of the PI-RADS lesion and persistently negative repeat mpMRI with the upgrading and persistence of the PI-RADS ≥3 lesion on the repeat mpMRI for the prediction of PC detection at the repeat biopsy, the sensitivity was 0.71, specificity 0.41, positive predictive value 0.28 and negative predictive value 0.82.

### 3.3. Initial and Follow-Up mpMRI of Two Patients with sPC Detection at Repeat Biopsy

**Figure 2:**
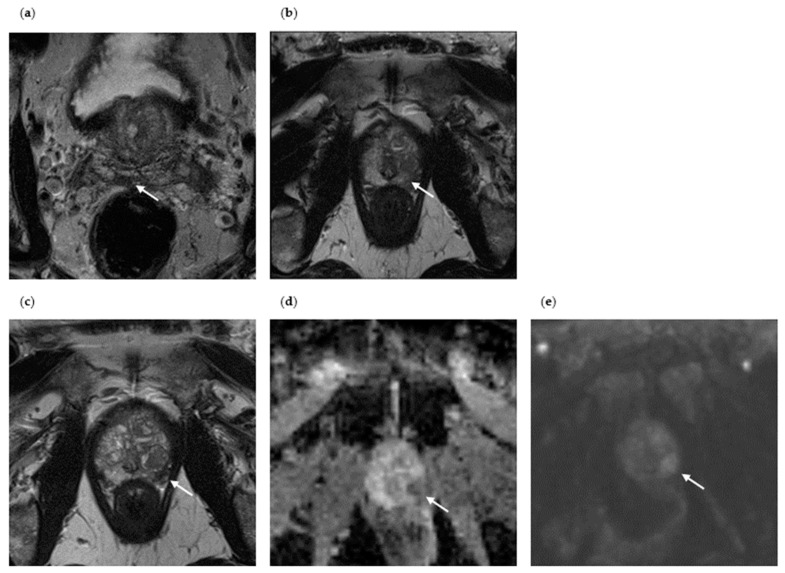
Axial mpMRI of a patient with sPC detection at repeat biopsy (Patient I). (**a**) Initial T2-weighted mpMRI without PC detection at initial biopsy (PI-RADS lesion 3, the suspicious lesion is marked with an arrow); (**b**) the PI-RADS 4 lesion visible in the repeat mpMRI does not yet present itself in the initial mpMRI (T2-weighted image); (**c**) repeat mpMRI of Patient I with sPC detection at repeat biopsy (new PI-RADS lesion 4, T2-weighted); (**d**) ADC decrease in the PI-RADS lesion 4 at repeat biopsy; (**e**) signal increase in the high b-value image at repeat biopsy.

**Figure 3:**
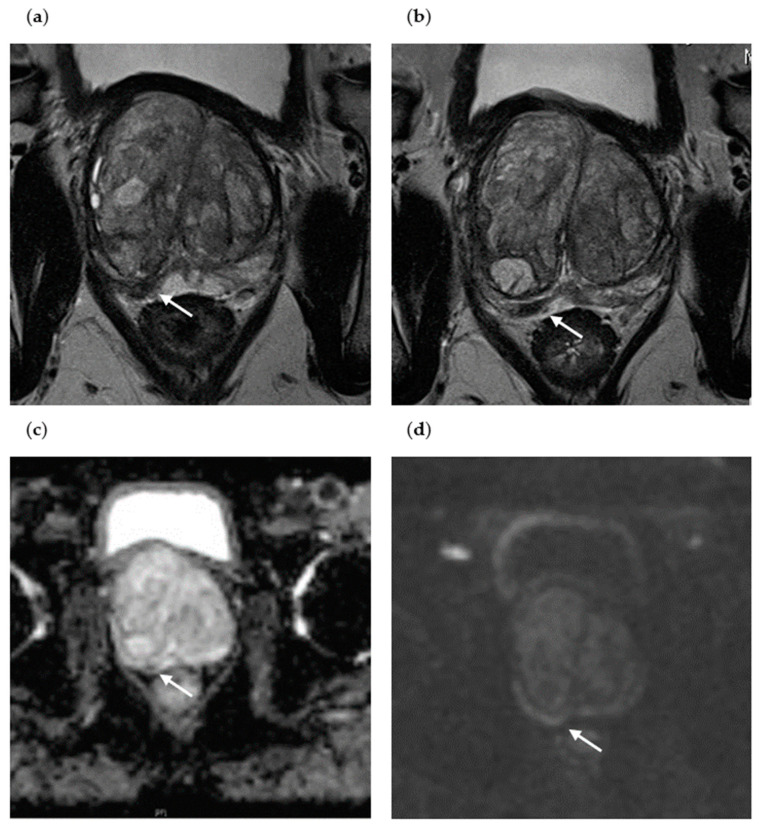
Axial mpMRI of a patient with sPC detection at repeat biopsy (Patient II). (**a**) Initial T2-weighted mpMRI of Patient II without PC detection at initial biopsy (PI-RADS lesion 4, the suspicious lesion is marked with an arrow); (**b**) repeat mpMRI of Patient II with sPC detection at repeat biopsy (persistent PI-RADS lesion 4, T2-weighted); (**c**) flat ADC decrease in the PI-RADS 4 lesion at repeat biopsy; (**d**) flat signal increase in the PI-RADS 4 lesion in the high b-value image at repeat biopsy.

The axial images of two patients in whom sPC was detected on the repeat MRI/TRUS-guided fusion prostate biopsy are shown in Figure 2 and Figure 3. Patient I is a 64-year-old male presenting with an initial PSA of 13.1 ng/mL (Figure 2). MpMRI revealed an overall PI-RADS 3 lesion at the right prostate base at the junction with the right seminal vesicle, which presented as a T2 hypointense focal lesion measuring 9 × 5 mm in the peripheral zone (PZpm), with focal diffusion restriction and embedded in diffuse contrast enhancement. Histopathological confirmation by initial biopsy ruled out malignancy. Within 2.5 years, the PSA level increased to 19.6 ng/mL. Repeat mpMRI revealed a new overall PI-RADS 4 lesion, a focal lesion in the left apical PZpm, T2 hypointense, with correlating ADC decrease, with signal enhancement in the high b-value image, and with early contrast agent enhancement. Histopathological confirmation by repeat mpMRI/TRUS-guided fusion biopsy revealed sPC with a GS 3 + 4 = 7a. In patient I, the detection of sPC on repeat biopsy is most likely a progressive or de novo tumor, as the PI-RADS 4 lesion on repeat mpMRI was not detected on the initial mpMRI (MRI progression).

Patient II is a 65-year-old patient presenting with an initial PSA value of 4.9 ng/mL (Figure 3). The initial mpMRI revealed a PI-RADS 4 lesion in the right central PZpm/pl, T2 hypointense, with flat ADC descent, with flat signal enhancement in the high b-value image, and with early contrast enhancement. Histopathological confirmation by initial biopsy ruled out malignancy. Within 1 year, the patient received a repeat mpMRI and a repeat MRI/TRUS-guided prostate fusion biopsy due to persistent suspicion of PC. The repeat mpMRI revealed a constant, suspicious focal PI-RADS 4 lesion in the right central PZpm/pl, T2 hypointense, similar to the previous examination, with flat ADC depression, with flat signal enhancement in the high b-value image, and with early contrast enhancement. Histopathological confirmation by repeat biopsy revealed sPC with a GS 4 + 3 = 7b. In patient II, the sPC detected on the repeat biopsy was most likely missed on the initial fusion biopsy due to a targeting error, possibly caused by the small size of the index lesion of 0.6 cm.

### 3.4. Initial and Follow-Up mpMRI of Two Patients without PC Detection at Repeat Biopsy

**Figure 4:**
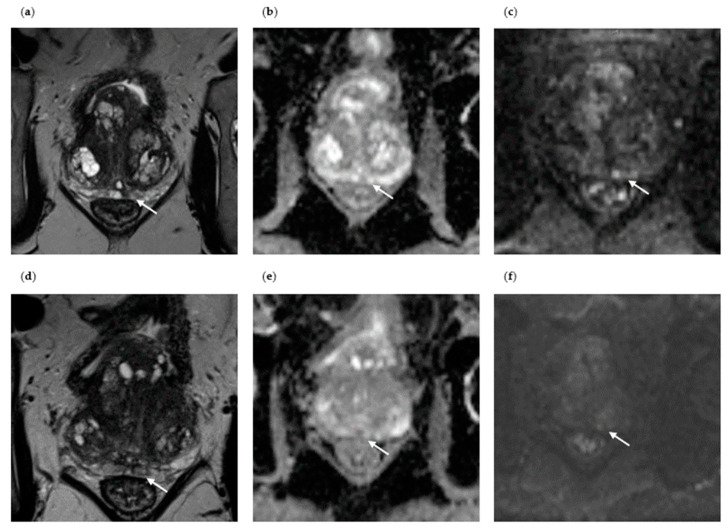
Axial mpMRI of a patient without PC detection at initial and repeat biopsy (Patient III). (**a**) Initial mpMRI of Patient III without PC detection at initial biopsy (PI-RADS lesion 4, T2-weighted image, the suspicious lesion is marked with an arrow); (**b**) marked ADC reduction in the PI-RADS 4 lesion at initial biopsy; (**c**) marked signal enhancement of the PI-RADS 4 lesion in the high b-value image at initial biopsy; (**d**) repeat mpMRI of Patient III without PC detection at repeat biopsy (PI-RADS lesion 3, T2-weighted image, focal lesion regressive T2 hypointense); (**e**) flat ADC reduction in the PI-RADS 3 lesion at repeat biopsy; (**f**) signal enhancement of the PI-RADS 3 lesion in the high b-value image at repeat biopsy.

**Figure 5:**
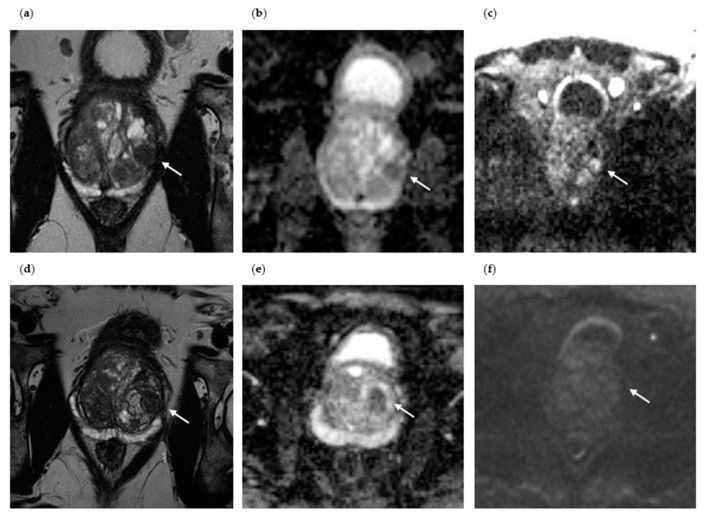
Axial mpMRI of a patient without PC detection at initial and repeat biopsy (Patient IV). (**a**) Initial mpMRI of Patient IV without PC detection at initial biopsy (PI-RADS lesion 3, T2-weighted image, the suspicious lesion is marked with an arrow); (**b**) moderate ADC reduction in the PI-RADS 3 lesion at initial biopsy; (**c**) discrete diffusion restriction of the PI-RADS 3 lesion in the high b-value image at initial biopsy; (**d**) repeat mpMRI of Patient IV without PC detection at repeat biopsy (PI-RADS score 2, T2-weighted image); (**e**) marked ADC reduction in the PI-RADS 2 lesion at repeat biopsy; (**f**) no signal enhancement of the PI-RADS 2 lesion in the high b-value image at repeat biopsy.

Figure 4 and Figure 5 show the axial images of two patients who had no evidence of malignancy on the initial and repeat MRI/TRUS-guided prostate biopsy. Patient III is a 63-year-old patient presenting with an initial PSA value of 4.6 ng/mL (Figure 4). MpMRI revealed a PI-RADS 4 lesion in the central left paramedian PZpm measuring 4 mm, T2 hypointense, with marked ADC reduction, and with marked signal enhancement in the high b-value image. Histopathological confirmation by initial biopsy ruled out malignancy, but showed myoglandular and fibromuscular hyperplasia, and chronic and localized florid prostatitis in the biopsy cores. Due to persistent suspicion of PC, a repeat mpMRI/TRUS-guided fusion biopsy was carried out one year later. The repeat mpMRI showed a downgrading of the pre-described PI-RADS 4 lesion to PI-RADS 3 and no new suspicious lesion. Compared to the initial imaging, the focal lesion in the central left PZpm, well delineated on the initial examination, was now regressive T2 hypointense, with flat ADC reduction, and with signal enhancement in the high b-value image. Repeat mpMRI/TRUS-guided fusion biopsy again excluded malignancy.

Patient IV is a 55-year-old patient with an initial PSA value of 12 ng/mL (Figure 5). MpMRI revealed a PI-RADS 3 lesion on the left side of the TZp, T2w hypointense, with discrete diffusion restriction on the B1500 image and moderate ADC subsidence. Histopathological confirmation by initial biopsy ruled out malignancy but showed focal chronic prostatitis. Due to a controlled rise in PSA to 18 ng/mL, a repeat biopsy was performed one year later. Repeat mpMRI showed a downgrading of the PI-RADS 3 lesion to a PI-RADS 2 lesion, with the appearance of a hyperplastic node and constant size. The lesion in the TZp now showed clear ADC depression, no reliable signal enhancement in the high b-value image and no early contrast enhancement. Repeat biopsy again excluded malignancy.

## 4. Discussion

The management of patients whose initial prostate biopsies are negative for PC is a common issue for urologists. MRI/TRUS-guided biopsies that do not result in a PC diagnosis may provide more reassurance than traditional TRUS-guided biopsies [25]. The effectiveness of TB is widely acknowledged, as MRI/TRUS fusion biopsies can detect more sPC than conventional 12-core TRUS-guided biopsies [33]. However, since small targeting errors of 1 to 2 mm can lead to missed sPC that is visible on MRI [34], patients might harbor PC despite a negative MRI/TRUS fusion biopsy. When PC is not detected on MRI/TRUS fusion biopsy despite a PI-RADS ≥ 3 lesion, the suspicion of missed PC remains. On the other side, the question is when to stop performing prostate repeat biopsies, since biopsies are associated with patient morbidity and biopsy-related costs [35]. As it is now suggested that mpMRI is utilized before prostate biopsy [13], the challenge for physicians is to determine which patients require an MRI/TRUS-guided repeat biopsy in cases where an initial MRI/TRUS-guided biopsy is negative and high clinical suspicion of PC is present. As most patients with an initial negative MRI/TRUS-guided biopsy are followed up with clinical examination and PSA testing, reports of repeat prostate biopsy are rare. In this study, we sought to analyze the incidence of PC and sPC in men who underwent a repeat MRI/TRUS-guided biopsy at our institution due to persistent suspicion of PC after an initial negative MRI/TRUS-guided biopsy.

Our analysis revealed that sPC was detected in 5% of men (3/58) on repeat MRI/TRUS-guided biopsy. This represents a significant improvement in an initial biopsy setting over the former standard 12-core TRUS-guided biopsy, which has a considerably lower diagnostic safety for the patient. In systematic TRUS-guided prostate biopsy, sPC is not an uncommon finding on repeat biopsy and the false negative rate for PC diagnosis is estimated to be 20–40% [36]. Thus, our data confirm that an MRI/TRUS-guided biopsy of the prostate improves the diagnostic safety of PC detection compared to the previous TRUS-guided approach. Comparing our results with recent studies, Barletta et al. [26] reported the sPC percentage to be 22% (15/68) in the repeat biopsy of a cohort of 68 patients with negative initial systematic and targeted prostate biopsy and PI-RADS ≥ 3 lesions on initial mpMRI). In a study of 31 patients who were offered a repeat TB after an initial negative TB, Venderink et al. [37] had detection rates for sPC of 20% (1/5), 11% (2/19), and 57% (4/7) for PI-RADS 3, 4, and 5 lesions, respectively. The detection rates for overall PC were 80% (4/5), 53% (10/19), and 86% (6/7), respectively. In a study by Pepe et al. [38], 256 patients with PI-RADS 3/4 lesions on mpMRI underwent early repeat prostate biopsy 6–9 months after an initial negative MRI/TRUS-guided fusion biopsy. The PC detection rate in repeat TB was 14% (36/256) and the sPC detection rate was 10% (26/256). In a study of Wallström et al. [39], patients with PI-RADS ≥ 3 lesions and a negative initial TB were offered repeat mpMRI and a repeat TB at 2 years after the initial biopsy. The PC and sPC detection rates at repeat biopsy were 42.1% (8/19) and 10.5% (2/19), respectively.

Comparing our results with these previous studies, it is noteworthy that our repeat biopsy sPC detection rate of 5% (3/58) is lower than those reported in previous literature. This might be because our initial MRI/TRUS-guided biopsy approach offers high diagnostic security, since a high number of prostate biopsy cores (median: 31 cores) are taken, and biopsies are performed in a high-volume hospital with many years of experience. An extensive sampling during prostate biopsy is particularly relevant in larger prostate volumes, since increased prostatic volume significantly reduces the chance of detecting PC [40]. Meanwhile, including the detection of GS 6 PC, our overall PC detection rate at repeat biopsy is 24% (14/58), which is comparable to previous studies.

In our study, there was no significant association between clinical parameters, PI-RADS lesions, or PI-RADS lesion downgrading on repeat MRI and the risk of PC detection at repeat biopsy that could be used to decide for or against repeat biopsy. The lack of statistical significance might be due to the small number of patients. We observed that none of the 19 patients whose PI-RADS score was downgraded on follow-up mpMRI had sPC detected on repeat biopsy. The data may indicate that there might be an association between mpMRI downgrading and sPC detection on repeat biopsy; however, the number of patients with sPC detection is very limited in our study. Comparable to our study design, Meng et al. [22] performed a follow-up on men with a PI-RADS 4 or 5 lesion and no PC detection in the initial biopsy. In their study, 45 men with an initial PI-RADS 4 or 5 lesion on mpMRI underwent repeat MRI, of whom 12 (27%) had persistent PI-RADS 4–5 abnormalities and 33 (73%) were downgraded to PI-RADS 1–3 lesions. The persistence of PI-RADS 4/5 on repeat mpMRI predicted a higher risk of missed PC, with 63% having PC on repeat MRI/TRUS-guided prostate biopsy, whereas a downgrading of PI-RADS score to 1–3 on repeat mpMRI was associated with a 15% PC detection rate on repeat MRI/TRUS-guided biopsy. Thus, the persistence or upgrade of a PI-RADS 4/5 lesion on repeat MRI strongly suggested that the initial biopsy may have missed the index lesion. In a recently published study by Stavrinides et al. [41], 58 patients with Likert 4 or 5 lesions on the initial mpMRI and a negative MRI-TB were followed up with a second mpMRI. In this study, the Likert 4 or 5 lesions persisted on the repeat mpMRI in only 5/58 men (9%). However, histopathological confirmation was lacking, with only 6/58 men undergoing repeat biopsy. Another study by Hauth et al. [42] described that 4.3% (2/46) of patients in their study had PI-RADS 3 lesions that progressed to PI-RADS 4 on repeat imaging, with subsequent detection of PC on the repeat biopsy. These findings emphasize the importance of a repeat mpMRI prior to repeat prostate biopsy in the setting of a negative initial biopsy, since downgrading or upgrading of the PI-RADS score might have value in patient selection for a repeat MRI/TRUS-guided biopsy.

Furthermore, as approximately 20% of primary PC is not visible on mpMRI [43], suspicion of PC may persist despite a negative MRI and an initial negative biopsy. This might especially be the case if the PSA level continues to rise rapidly after an initial negative biopsy, or if the first biopsy was difficult to perform due to a large prostate size. In our study, only some of the seven men with an initial negative mpMRI (PI-RADS ≤ 3 lesion) had a negative MRI on the repeat biopsy (Table 2), but none of these seven men with an initial negative mpMRI (0/7) had PC detected on the repeat biopsy (Table 1). The number of patients in our study is limited; however, these data might contribute to helping inform patients and clinicians in shared decision making in case of a negative initial mpMRI and prostate biopsy, and persistent clinical suspicion of PC, to potentially safely omit a prostate re-biopsy. Our findings are similar to those of Lo et al. [44], who found that men with a negative biopsy and negative MRI findings had an extremely low risk of sPC at a median of 6.7 years, suggesting that these men may not require a repeat biopsy in the first few years.

The available literature with mostly small study sizes demonstrates the need for future prospective, multi-center trials to better manage patients with negative prostate biopsy results in the era of initial MRI/TRUS-guided biopsy. Future work should concentrate on defining a surveillance and intervention strategy for men with an initial negative MRI/TRUS-guided prostate biopsy, such as repeat mpMRI in case of suspicious PI-RADS lesions. A recent review suggested patient stratification according to the overall PI-RADS lesion on initial mpMRI for the follow-up of patients with an initial negative prostate biopsy [23]: For PI-RADS ≤ 3 lesions, clinical controls with PSA and follow-up mpMRI at 6–12 months was suggested. For PI-RADS 4 lesions, clinical controls and follow-up mpMRI at 3–6 months was recommended to guide decision on a follow-up biopsy, while for PI-RADS 5 lesions standard follow-up biopsy was advised. A decision against a prostate re-biopsy requires a robust surveillance program similar to an active surveillance protocol. In the event of an increase in PSA levels or PI-RADS lesion upgrade, a repeat biopsy should be reconsidered.

Over the next few decades, clinical and genetic markers will have the potential to improve the ability to determine the necessity for repeat biopsy [45]. Epigenomic modifications, particularly DNA methylation at the 5′-carbon of cytosine residues (5-mC), have been extensively studied in cancer. Promoter hypermethylation and consequent gene silencing take place in several genes in PC, including *GSTP1*, *RASSF1*, *APC*, *CCND2*, and *PITX2*. Their 5-mC levels have shown high sensitivity and specificity in distinguishing PC from benign tissue [46,47,48,49,50,51,52]. A combination of *GSTP1*, *RASSF1*, and *APC* analysis showed better performance than histological evaluation of biopsy samples, identifying PC in 62% and 68% of histologically negative biopsies in two separate large studies (analyzing 498 and 350 biopsies, respectively) [47,49]. Therefore, 5-mC biomarkers may potentially overcome the limitations of biopsy sampling and aid in the detection of PC in histologically occult biopsy cores [53]. In addition, studies have suggested that the Prostate Cancer Antigen 3 (PCA3) score and the Prostate Health Index (PHI) could improve the accuracy of predicting the presence of PC at repeat biopsy [54].

Our study has several limitations. First, our results were obtained in a single tertiary care center with extensive experience in mpMRI and prostate fusion biopsy. The use of mpMRI in PC detection requires a learning curve for the entire team; thus, the generalizability of our results to a real-world setting must be made with caution [13,55,56].

In addition, our study is subject to all the potential biases that are expected in a retrospective study design. We acknowledge that not all patients with an initial negative biopsy and clinical suspicion of PC have received a planned re-biopsy and that the number of men who underwent a repeat biopsy is limited. In addition, we did not have access to the information on whether patients have had a repeat mpMRI or prostate biopsy at another institution. Due to the small sample size, differences between the groups might well not have been detected. Nevertheless, our study corresponds to the real-life setting, since there are currently no international recommendations on who and when to re-biopsy after a negative MRI/TRUS-guided biopsy. These circumstances are consistent with the situation described in a recent review by Grivas et al., in which the authors were confronted with the small number of studies on repeat biopsy after an initial negative MRI/TRUS-guided prostate biopsy. After study protocol revision, nine studies were included in the review, seven of which were conducted with <50 study patients [23].

Furthermore, it should be noted that in addition to lesions missed on the initial biopsy due to misregistration, the sPC detection rate of repeat targeted biopsy can be affected by newly detected lesions due to disease progression. Newly developed PC or PC newly visible on MRI should be especially considered if there is a long time interval between the initial and repeat biopsy.

Our study distinguishes itself, as our approach of TB and SB at initial and repeat biopsy provides a very high diagnostic security of the presence of PC, having been correlated to RP histopathology [31]. However, it is important to note that the very high number of biopsy cores is above average for most institutions and thus makes our findings less generalizable.

Several studies have shown that some sPC is missed in fusion TB, and that the combination of SB and TB detects more sPC than TB alone [57]. It is up for debate whether a repeat SB is necessary after an initial negative MRI/ultrasound-guided biopsy and how high the diagnostic certainty of prostate biopsy should be. To date, there is a lack of evidence and strong international recommendations to support a specific follow-up strategy in men with a negative mpMRI/TRUS-guided biopsy. A recent potential solution to overcome the problem of fusion errors may be the use of so-called ‘target saturation’, i.e., the application of a median of 8–10 cores to the lesion and penumbra [58,59]. This is also supported by the PI-RADS v2.1 steering committee [60].

Finally, our reference standard for PC detection is SB and TB instead of RP, as expected in the setting of PC detection in an at-risk population. However, the method of combined MRI-TB and SB has previously been validated at our institution by correlation with RP specimen [31].

## 5. Conclusions

The patients in our study cohort with an initial negative mpMRI/TRUS-guided prostate biopsy had a high likelihood of not having sPC (95%) at repeat biopsy after a median of 18 months. None of the patients with sPC detected on the repeat biopsy had PI-RADS score downgrading on the follow-up mpMRI, which may indicate that there is an association. Due to the small cohort size, further research is recommended.

## Figures and Tables

**Table 1:** Patient characteristics, magnetic resonance imaging findings, and biopsy cores at initial biopsy for patients with and without prostate cancer detected at repeat biopsy. DRE = digital rectal examination; IQR = inter-quartile range; PC = prostate cancer; PI-RADS = Prostate Imaging Reporting and Data System; PSA = prostate-specific antigen; yr = year.

	No PC in Re-Biopsy	PC in Re-Biopsy	*p* Value
Men included in group, *n*	44	14	
Men with significant PC in re-biopsy, *n* (%)	0	3	
Men with insignificant PC in re-biopsy, *n* (%)	0	11	
Age at initial biopsy, yr, median (IQR)	59 (54–65)	59 (52–64)	0.81
PSA level at initial biopsy, ng/mL, median (IQR)	6.6 (4.9–8.5)	6.8 (4.4–9)	0.58
Suspicious DRE finding at initial biopsy (≥T2), *n* (%)	5 (11)	2 (14)	0.77
Prostate volume at initial biopsy, mL, median (IQR)	50 (40–70)	57 (48–65)	0.94
PSA density at initial biopsy, ng/mL^2^, median (IQR)	0.13 (0.08–0.19)	0.14 (0.07–0.17)	0.78
Biopsy cores per patient at initial biopsy, median (IQR)	30 (27–33)	32 (29–34)	0.64
Overall PI-RADS 1–2 lesion at initial biopsy, *n* (%)	7 (16)	0 (0)	0.11
Overall PI-RADS 3–5 lesion at initial biopsy, *n* (%)	37 (84)	14 (100)	0.11
Overall PI-RADS 3 lesion at initial biopsy, *n* (%)	11 (25)	8 (57)	0.03
Overall PI-RADS 4 lesion at initial biopsy, *n* (%)	21 (48)	5 (36)	0.43
Overall PI-RADS 5 lesion at initial biopsy, *n* (%)	5 (11)	1 (7)	0.65
Location of the lesion in the peripheral zone, *n* (%)	28 (76)	12 (86)	0.44
Location of the lesion in the transitional zone, *n* (%)	9 (24)	2 (14)	0.44
Volume of the lesion, mL, median (IQR)	0.31 (0.19–0.53)	0.26 (0.23–0.32)	0.33

**Table 2:** Patient characteristics, magnetic resonance imaging findings, and biopsy cores at repeat biopsy for patients with and without prostate cancer detection at repeat biopsy. DRE = digital rectal examination; IQR = inter-quartile range; mpMRI = multiparametric magnetic resonance imaging; PC = prostate cancer; PI-RADS = Prostate Imaging Reporting and Data System; PSA = prostate-specific antigen; yr = year.

	No PC in Re-Biopsy	PC in Re-Biopsy	*p* Value
Men included in group, *n*	44	14	
Men with significant PC in re-biopsy, *n* (%)	0	3	
Men with insignificant PC in re-biopsy, *n* (%)	0	11	
Months after initial biopsy, median (IQR)	18 (11–25)	17 (9–22)	0.50
Age at repeat biopsy, yr, median (IQR)	61 (57–69)	60 (56–67)	0.70
PSA level at repeat biopsy, ng/mL, median (IQR)	9.0 (6.0–12.0)	7.0 (5.3–9.7)	0.20
Suspicious DRE finding at repeat biopsy (≥T2), *n* (%)	3 (7)	1 (7)	0.97
Prostate volume at repeat biopsy, mL, median (IQR)	64 (48–92)	54 (40–78)	0.18
PSA density at repeat biopsy, ng/mL^2^, median (IQR)	0.14 (0.078–0.19)	0.14 (0.07–0.20)	0.56
Biopsy cores per patient at repeat biopsy, median (IQR)	31 (27–35)	31 (25–34)	0.61
Overall PI-RADS 1–2 lesion at repeat biopsy, *n* (%)	7 (16)	4 (29)	0.29
Overall PI-RADS 3–5 lesion at repeat biopsy, *n* (%)	37 (84)	10 (71)	0.29
Overall PI-RADS 3 lesion at repeat biopsy, *n* (%)	19 (43)	3 (21)	0.14
Overall PI-RADS 4 lesion at repeat biopsy, *n* (%)	14 (32)	5 (36)	0.79
Overall PI-RADS 5 lesion at repeat biopsy, *n* (%)	4 (9)	2 (14)	0.58
Downgrading of PI-RADS lesion on repeat mpMRI, *n* (%)	15 (34)	4 (29)	0.70
Upgrading of PI-RADS lesion on repeat mpMRI, *n* (%)	7 (16)	2 (14)	0.88
Constant PI-RADS ≥ 3 lesion on repeat mpMRI, *n* (%)	19 (43)	8 (57)	0.36
Persistent negative repeat mpMRI (PI-RADS < 3), *n* (%)	3 (7)	0	0.32
Location of the lesion in the peripheral zone, *n* (%)	24 (65)	8 (80)	0.36
Location of the lesion in the transitional zone, *n* (%)	13 (35)	2 (20)	0.36
Volume of the lesion, mL, median (IQR)	0.37 (0.23–0.76)	0.15 (0.11–0.21)	0.93

## Data Availability

The data presented in this study are available on request from the corresponding author.

## Supplementary Materials

The following supporting information can be downloaded at: https://www.mdpi.com/article/10.3390/diagnostics13101761/s1.

## Abbreviations

ADC	apparent diffusion coefficient
AZ	anterior zone
DRE	digital rectal examination
DWI	diffusion-weighted images
GS	Gleason score
IQR	interquartile range
ISUP	International Society of Urological Pathology
mpMRI	multiparametric magnetic resonance imaging
PC	prostate cancer
PCA3	prostate cancer antigen 3
PI-RADS	Prostate Imaging Reporting and Data System
PHI	Prostate Health Index
PSA	prostate-specific antigen
PZ	peripheral zone
RP	radical prostatectomy
SB	systematic saturation biopsy
sPC	significant prostate cancer
TB	targeted fusion biopsy
TRUS	transrectal ultrasound
TZ	transition zone
5-mC	5′-carbon of cytosine residues
