# Supplementary material for: Detection Rate of Prostate Cancer in Repeat Biopsy after an Initial Negative Magnetic Resonance Imaging/Ultrasound-Guided Biopsy
Source: Diagnostics (Basel). 2023 May 17;13(10):1761. doi: 10.3390/diagnostics13101761 (PMC10217194; doi:10.3390/diagnostics13101761)
Supplement: Supplementary file 1 [file diagnostics-13-01761-s001.zip › diagnostics-2376505-supplementary.pdf]

Supplementary Material S1

Prostate MRI Protocol

Sequence	Orien-tation	TE (ms)	TR (ms)	Section Thick-ness (mm)	FOV read (mm)/FoV Phase %	Base (mm)/ Phase % (section%) Resolution	Avgs.	Phase Encod-ing	Additional Features	Additional Features
T2 blade	sag	105	5000	3	200/100%	320/100%	1	F-H	Blade Coverage 81%	
T1 tse dixon	cor	13	773	4	360/90%	320/100%	1	F-H	TF 5	
T2 tse	tra	145	8080	3	200/100%	320/100%	3	R-L	TF 27	
T2 tse	cor	145	8000	3	200/100%	320/80%	3	F-H	TF 19	
epi_diff SPAIR	tra	48	3300	3	280/74%	140/100%	2,2,4,6	A-P	phase partial Fourier 7/8	b = 50/500/ 1000/1500
dynamic DCE										
T1_twist_DCE_ Dyn	tra	4.45	3	3	300/87%	256/85%/65%	1	R-L	35 meas./ Temporal Resolution 5 s	30 sections per slab
T1_vibe_dixon	tra	2.4/3. 69	5.4	1.2	380/68%	384/80%/50%	1	A-P	W-images/F-images	224 sections per slab

Note.—General preparations: MRI-coil: body array coil; patients placed supine. TE = echo time, TR = repetition time, FOV = field of view, TSE = turbo-spin-echo, DCE = dynamic contrast enhanced, SPAIR = spectral adiabatic inversion recovery, FOV = field of view, TF = turbo factor, tra/sag/cor = transverse/sagittal/coronal.
